# Long-Term Clinical Outcomes of Fractional Flow Reserve-Guided Coronary Artery Revascularization in Chronic Kidney Disease

**DOI:** 10.3390/jpm12010021

**Published:** 2022-01-01

**Authors:** Chien-Boon Jong, Tsui-Shan Lu, Patrick Yan-Tyng Liu, Jeng-Wei Chen, Ching-Chang Huang, Hsien-Li Kao

**Affiliations:** 1 Department of Internal Medicine, National Taiwan University Hospital, Hsin-Chu Branch, Hsin-Chu 300195, Taiwan; jgboon0407@gmail.com; 2 School of Medicine, College of Medicine, National Taiwan University, Taipei 100233, Taiwan; b85401044@ntu.edu.tw (C.-C.H.); hsienli_kao@yahoo.com (H.-L.K.); 3 Department of Mathematics, National Taiwan Normal University, Taipei 116059, Taiwan; evatslu@gmail.com; 4 Division of Cardiology, Department of Internal Medicine and Cardiovascular Center, National Taiwan University Hospital, Taipei 100225, Taiwan; patrickytliu@gmail.com; 5 Graduate Institute of Clinical Medicine, College of Medicine, National Taiwan University, Taipei 100233, Taiwan; 6 Department of Surgery, National Taiwan University Hospital, Taipei 100225, Taiwan

**Keywords:** chronic kidney disease, clinical outcome, fractional flow reserve, revascularization

## Abstract

Fractional flow reserve (FFR)-guided percutaneous coronary intervention has shown favorable long-term clinical outcomes. However, limited data exist evaluating the FFR assessment among the chronic kidney disease (CKD) population. The aim of this study was to evaluate the long-term clinical outcomes of FFR-guided coronary revascularization in patients with CKD. A total of 242 CKD patients who underwent FFR assessment were retrospectively analyzed. Patients were divided into two groups: revascularization (FFR ≤ 0.80) and non-revascularization (FFR > 0.80). The primary endpoint was the composite of cardiac death, non-fatal myocardial infarction, and target vessel failure (TVF). The key secondary endpoint was TVF. The Cox regression model was used for risk evaluation. With 91% of the ischemic vessels revascularized, the revascularization group had higher risks for both the primary endpoint (adjusted hazard ratio [aHR]: 2.06; 95% confidence interval [CI], 1.07–3.97; *p* = 0.030) and key secondary endpoint (aHR: 2.19, 95% CI: 1.10–4.37; *p* = 0.026), during a median follow-up of 2.9 years. This result was consistent among different CKD severities. In patients with CKD, functional ischemia in coronary artery stenosis was associated with poor clinical outcomes despite coronary revascularization.

## 1. Introduction

The global burden of chronic kidney disease (CKD) has increased and is accompanied by an important risk factor for cardiovascular disease [1]. CKD affects 1 in 10 adults of different races in different countries [2]. Patients with CKD have a higher prevalence of cardiac mortality and cardiovascular disease [3,4]. Therefore, screening for coronary artery disease (CAD) and optimizing coronary revascularization are important issues in the CKD population. Recently, the International Study of Comparative Health Effectiveness with Medical and Invasive Approaches–Chronic Kidney Disease (ISCHEMIA-CKD) trial showed no benefit of an initial invasive strategy in stable CAD with advanced CKD and moderate to severe ischemia compared with initial medical therapy [5]. However, the screening tool in this trial was noninvasive and involved mainly nuclear imaging studies, which provided suboptimal predictive value for obstructive CAD in CKD [6].

The fractional flow reserve (FFR) is an invasive physiologic index, defined as the ratio of the mean distal pressure (distal to the stenotic lesion, Pd) to the mean proximal pressure (aortic pressure, Pa) in the coronary artery while the maximal hyperemic flow is achieved [7]. Currently, FFR is highly recommended to assess the hemodynamic relevance of intermediate-grade stenosis of the epicardial coronary artery in the guideline of myocardial revascularization [8]. FFR-guided percutaneous coronary intervention (PCI) was shown to be superior to angiographic-guided PCI in terms of follow-up risk of major adverse cardiac events (MACEs) for up to 2 years [9]. Patients who underwent FFR-guided PCI had a non-inferior MACE outcome compared to patients with hemodynamic insignificant stenosis with medical therapy alone and a superior MACE outcome compared to patients with significant hemodynamic stenosis with medical therapy alone [10]. Nevertheless, there was only a small portion of the CKD population included in these clinical trials. In patients with CKD, the coronary flow reserve (CFR) was declined in mild CKD and at its nadir in advanced CKD [11]. The low CFR was highly correlated with microvascular dysfunction, which may have caused suboptimal hyperemia during FFR assessment and influenced the accuracy of the FFR value. Consequently, the clinical benefit of FFR assessment in patients with CKD is uncertain. This study aimed to evaluate the efficacy of FFR-guided coronary artery revascularization in patients with CKD, including those with end-stage kidney disease. Owing to the discordance of the resting physiologic index and FFR in CKD, the accuracy and optimal cutoff FFR to predict clinical outcomes were calculated.

## 2. Materials and Methods

### 2.1. Study Design and Subjects

**Figure 1:**
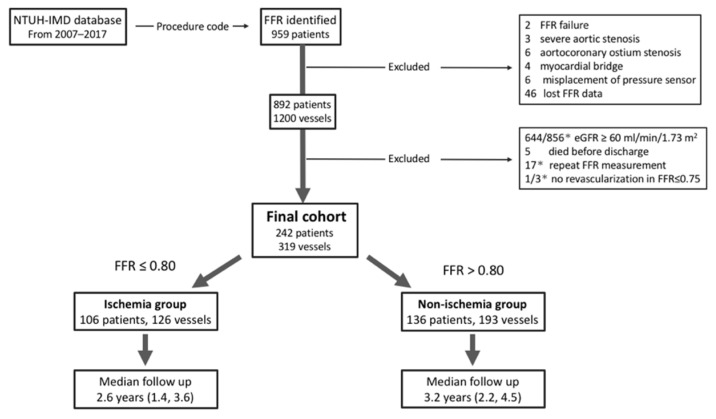
Diagram of patient flow. The final cohort consists of 242 patients; 44% have functional ischemia with FFR ≤ 0.8. * Counts as per vessel, otherwise count as per person. Abbreviations: eGFR, estimated glomerular filtration rate; FFR, fractional flow reserve; NTUH-IMD, National Taiwan University Hospital integrated medical database.

This was a retrospective study conducted in National Taiwan University Hospital and its affiliated Hsinchu Branch. The study subjects were selected according to medical claims for a pressure-monitoring guidewire (Certus or Aeris pressure wire; St. Jude Medical Inc., St. Paul, MN, USA). The diagram of patient flow is shown in Figure 1. After a medical record review, 67 patients were excluded due to the following criteria: severe aortic stenosis, aortocoronary ostium stenosis, myocardial bridge, misplacement of the pressure sensor, or incomplete FFR data. In addition, 644 patients with normal renal function, 5 patients who died before discharge, and 1 patient who did not show any coronary revascularization (FFR ≤ 0.75) were excluded. The final CKD (defined as an estimated glomerular filtration rate <60 mL/min/1.73 m^2^) cohort enrolled 242 patients with 319 vessels (Figure 1). The formula for modification of diet in renal disease was used to calculate the estimated glomerular filtration rate. The severity of CKD was characterized as mild CKD (Stage 3a), moderate to advanced CKD (Stage 3b–5), or dialysis-dependent CKD, which were defined as an estimated glomerular filtration rate of 45–60 mL/min/1.73 m^2^, and an estimated glomerular filtration rate of 0–45 mL/min/1.73 m^2^, and dependence on dialysis, respectively.

### 2.2. Data Collection and Ethical Approval

The study population data were mainly extracted from the integrated medical data- base of the National Taiwan University Hospital. The details of this database had been introduced previously [12]. Patients in this database can be linked to the Taiwan National Death Registry by patient identification numbers to obtain the date and cause of death [13]. To optimize the accuracy of information from the database and the Taiwan National Death Registry, chart reviews were conducted by two experienced cardiologists (C.B.J. & J.W.C.) with a special focus on causes of readmission due to cardiovascular events. This study was approved by the institutional review board (No. 201910092RINC) of the National Taiwan University Hospital, Taipei, Taiwan, and the requirement for informed consent was waived. All methods were performed in accordance with the Declaration of Helsinki and regulations.

### 2.3. Outcomes and Follow-Up

The primary endpoint was the composite outcome of cardiac death, non-fatal myocardial infarction, and target vessel failure (TVF). The key secondary endpoint was target vessel failure. The additional secondary endpoints were the components of the primary endpoint, all-cause death, and composite outcome of cardiac death and non-fatal myocardial infarction. The definition of clinical events was the same as previously described [14]. TVF was defined as target vessel revascularization driven by clinical ischemia or myocardial infarction. The beginning of the follow-up period was the date of FFR assessment, while the end was the date of death, the date of a clinical event, or 4 October 2019, whichever occurred first.

### 2.4. Statistical Methods

The tabular statistical method (e.g., means shown with one standard error for continuous variables or counts with percentages for categorical variables) was used to present the baseline characteristics, and the patients were divided into two groups: the revascularization group (FFR ≤ 0.8) and the non-revascularization group (FFR > 0.8). We compared differences between the two groups using the Wilcoxon rank-sum test for continuous variables and the chi-squared test for categorical variables. We first checked the assumption of the proportional hazards and then carried out the primary inferential analyses for the incidences of all clinical outcomes, conditional on the exploratory variables (FFR, age, sex, end-stage kidney disease, heart failure, left main or triple-vessel disease, target vessel with left main or ostium/proximal part of the left anterior descending artery, hemoglobin, and the interaction term of end-stage kidney disease and hemoglobin) and relying on the Cox proportional hazards model accompanied by 95% confidence limits. To assess the discriminative performance of the FFR, the time-dependent receiver operating characteristic curves were computed at specific time points using the inverse probability of censoring weighting method [15]. All statistical analyses were performed using SAS software version 9.4 (SAS Institute, Cary, NC, USA).

## 3. Results

### 3.1. Patient and Vessel Characteristics

The characteristics of the patients and vessels are summarized in Table 1. Of the 242 patients, one-third were female, 90% had vessels with intermediate stenosis, and 44% had vessels with an FFR ≤ 0.8. Most patients had stable CAD, and 27% of patients were receiving long-term dialysis. Compared with the non-revascularization group (FFR > 0.8), the revascularization group (FFR ≤ 0.8) had more comorbidities of heart failure and dialysis-dependent CKD, more extensive CAD, and more target vessels of either the left main or ostium/proximal part of the left anterior descending artery, which represent a larger threat to the myocardium. Ten patients with 12 vessels (9.5% of the revascularization group) with an FFR interval of 0.77–0.80 (median: 0.80 ± 0.0025) received medical therapy only. Of the remaining 114 vessels in the revascularization group, most patients received PCI and only 2% underwent coronary artery bypass grafting surgery. Nearly two-thirds of patients with PCI received either a drug-eluting stent or bioresorbable vascular scaffold implantation. The FFR-guided management strategies are summarized in Table 2. The median follow-up time was shorter in the revascularization group than in the non-revascularization group (2.6 years vs. 3.2 years).

### 3.2. Clinical Outcomes

**Figure 2:**
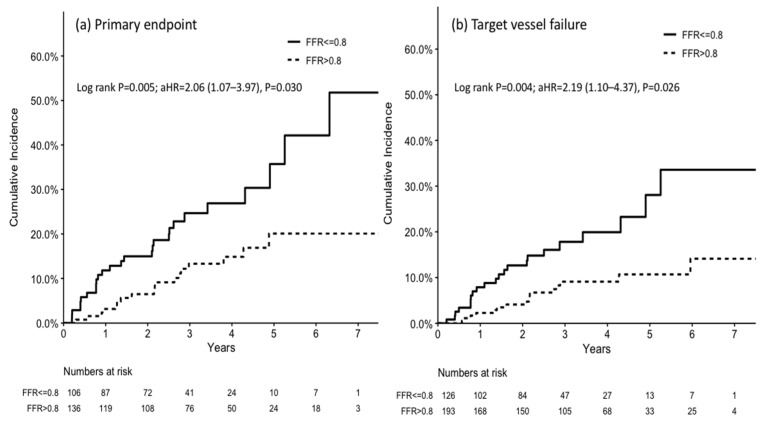
Kaplan–Meier curves for (**a**) the primary endpoint and (**b**) target vessel failure. The cumulative incidences of both the primary endpoint (a composite outcome of cardiac death, non-fatal myocardial infarction, and target vessel failure) and target vessel failure were higher in the revascularization (FFR ≤ 0.8) group than in the non-vascularization (FFR > 0.8) group. Abbreviations: aHR, adjusted hazard ratio; FFR, fractional flow reserve.

The primary endpoint rate of the revascularization group was higher than that of the non-revascularization group (25.5% vs. 13.2%; adjusted hazard ratio[aHR], 2.06; 95% confidence interval [CI], 1.07–3.97; *p* = 0.030) at a median follow-up of 2.9 years. Additionally, 90.5% of the patients in the revascularization group had received coronary revascularization (Table 3). This result was consistent when stratified according to CKD severity (Table 4). The target vessel failure rate was also higher in the revascularization group than in the non-revascularization group (17.5% vs. 8.3%; aHR, 2.19; 95% CI, 1.10–4.37; *p* = 0.026). The other secondary endpoints did not differ significantly between the two groups (Table 3). The Kaplan–Meier curves for the primary endpoint and key secondary endpoint are shown in Figure 2.

### 3.3. The Predicted Value and Best Cutoff of FFR to Predict Clinical Outcome

The area under the time-dependent receiver operating characteristic curve was 0.70 when using FFR to predict the primary endpoint. The best cutoff value of FFR in predicting the primary endpoint at 1 year was 0.78, with an area under the time-dependent receiver operating characteristic curve of 0.72, and an accuracy of 70% (sensitivity 75%, specificity 69%, negative predictive value 98%).

## 4. Discussion

To our knowledge, this is the first study to evaluate the relationship between coronary invasive physiologic index and cardiovascular outcomes in a CKD population, as well as in patients with dialysis-dependent CKD. This study showed that functional ischemia in coronary stenosis was associated with a higher risk of the composite outcome of cardiac death, non-fatal myocardial infarction, and ischemia-driven revascularization. This result was consistent among different CKD severities. In addition, the accuracy of FFR in predicting this composite outcome was acceptable.

In the Fractional Flow Reserve Versus Angiography for Multivessel Evaluation 2 study, the FFR-guided PCI strategy was associated with lower MACE outcome rates after 5 years than those of patients with functional ischemia who received medical therapy alone. FFR-guided PCI also showed a non-inferior MACE outcome compared to patients with hemodynamic significant stenosis and medical therapy alone [10]. However, only 2% of the CKD population was reported in a previous trial. Our study showed a higher MACE outcome rate in the functional ischemia group than that in the non-ischemia group, and this result was consistent among different CKD severities. The failure of coronary revascularization in lowering MACE could have been due to a few reasons. First, residual ischemia, since diffuse and tandem lesions were prominent in the functional ischemia group; routine post-PCI FFR assessment should be considered in such cases to exclude the possibility of residual ischemia [16,17]. Notably, only 39% of post-PCI FFR was available in the revascularization group in this study (data not shown). Moreover, coronary flow reserve assessment can also be considered due to the high prevalence of microvascular dysfunction in the CKD population, which might influence the accuracy of FFR in such conditions [11,18]. Second, accelerated atherosclerosis was not uncommon in advanced CKD [19]; thus, a second-generation drug-eluting stent or coronary artery bypass grafting surgery should be considered instead of bare-metal stent implantation in the revascularization strategy. One-third of ischemic vessels in this study received either bare-metal stent implantation or balloon angioplasty alone, which may have contributed to the unfavorable outcome in the ischemic group (Supplemental Table S1). Furthermore, optimization of the cardiovascular risk modification and slowing the progression of renal disease in the mild stages of CKD were suggested. Accelerated atherosclerosis is usually developed in advanced CKD, and there is a lack of effective plaque modifiers in this stage [19].

In the ISCHEMIA-CKD trial, only 50% of revascularization was performed in the invasive group, and approximately one-quarter of the patients in the invasive group had non-obstructive coronary disease. These results imply that either the accuracy of noninvasive physiology tests or the high prevalence of microvascular disease were the concern in advanced CKD [5]. Similarly, the accuracy of FFR assessment to evaluate the hemodynamic significance of coronary stenosis in the CKD population also requires further data, since submaximal hyperemia may occur with microvascular dysfunction. The instantaneous wave-free ratio, a non-hyperemic physiologic index that is less independent of microvascular conditions, has been found to be inconsistent with FFR results in patients undergoing hemodialysis [20]. Our study group recently found another invasive physiologic index, nitroglycerine-induced acute drop of Pd/Pa, to be disproportionate to the FFR value while deteriorating renal function [21]. Based on the limited data, we suspect that the optimal cutoff of FFR, instantaneous wave-free ratio, or nitroglycerine-induced acute drop of Pd/Pa may differ between CKD and non-CKD populations. This study found that the best FFR cutoff value in predicting MACE outcomes was 0.78. In contrast, Johnson et al. [22] stated that the optimal FFR threshold for MACE outcome was 0.67 in a patient-level meta-analysis. However, in the diabetes subgroup, which usually had a higher incidence of microvascular dysfunction and poorer MACE outcomes, the threshold increased to 0.79, which was similar to the threshold in the CKD population in this study [22]. However, ours was a small study, and larger prospective studies are needed to evaluate the accuracy and cutoff of FFR values to predict clinical outcomes in the CKD population.

There were several limitations in our study. First, this was a retrospective observational study that provided only associative, not causative, evidence; hence, our findings should be interpreted with caution. Second, selection bias, residual unmeasured confounding factors, and survival bias might exist because only survived, discharged patients were included. Third, cardiac death contributed to one-quarter of overall mortality; this rate was lower than that in a previous report [5]. To draw nearer to the truth, we reviewed the medical charts and reconfirmed the report from the Taiwan National Death Registry.

## 5. Conclusions

FFR is a reliable index to guide coronary revascularization strategy in the CKD population, as well as in patients with dialysis-dependent CKD, since it stratifies clinical outcomes. However, the FFR-guided coronary revascularization strategy did not modulate the unfavorable outcome of ischemia in this study, probably partly explained by a high percentage of patients treated with bare-metal stent or balloon angioplasty alone.

## Figures and Tables

**Table 1:** Baseline and target vessel characteristics of patients with CKD who received FFR examination at NTUH, stratified by FFR ≤ 0.8 (revascularization) and FFR > 0.8 (non-revascularization).

Demographic	Revascularization (*n* = 106 Patients)	Non-Revascularization (*n* = 136 Patients)	*p*-Value
Age	69.3 ± 11.4	72.3 ± 10.5	0.050
Sex (female), n (%)	29 (27.4)	55 (40.0)	0.034
Body mass index	26.0 ± 3.9	25.7 ± 4.1	0.475
Current smoker, n (%)	19 (17.9)	18 (13.3)	0.326
Comorbidity, n (%)			
Hypertension	90 (84.9)	119 (87.5)	0.560
Diabetes mellitus	64 (60.4)	71 (52.2)	0.204
Heart failure	34 (32.1)	27 (19.9)	0.030
LVEF ≤ 50%	21 (20.6)	19 (14.7)	0.243
Previous myocardial infarction	5 (4.7)	3 (2.2)	0.278
History of coronary artery bypass grafting	6 (5.7)	9 (6.6)	0.759
CKD status			
Mild CKD	43 (40.6)	62 (45.6)	0.434
Moderate to advanced CKD	23 (21.7)	48 (35.3)	0.021
Dialysis-dependent CKD	40 (37.7)	26 (19.1)	0.001
Distribution of arterial disease, n (%)			
Left main disease	15 (14.2)	7 (5.1)	0.004
CAD single-vessel disease	12 (11.3)	52 (38.2)	<0.0001
CAD double-vessel disease	41 (38.7)	34 (25.0)	0.015
CAD triple-vessel disease	53 (50.0)	50 (36.8)	0.055
Peripheral arterial disease	16 (15.1)	15 (11.0)	0.348
History of stroke	13 (12.3)	13 (9.6)	0.500
Clinical presentation *, n (%)			
Acute coronary syndrome	19 (15.1)	19 (9.8)	0.165
Stable CAD	106 (84.1)	174 (90.2)	
Heart failure	1 (0.8)	0 (0)	
Lab data			
Estimated glomerular filtration rate, ml/min/1.73 m^2^	46.7 ± 10.7	45.0 ± 11.9	0.536
Hemoglobin, g/dL	12.0 ± 2.2	12.6 ± 2.1	0.041
HbA1c, mmol/mol	7.0 ± 1.7	7.1 ± 1.8	0.858
LDL, mg/dL	96.6 ± 37.1	94.0 ± 32.9	0.840
Target vessel *, n(%)			
Left main or ostium/proximal part of left anterior descending artery	48 (38.1)	35 (18.1)	0.0004
Other coronary arteries	78 (61.9)	158 (81.9)	
Extent of atherosclerosis *, n (%)			
Diffuse ^†^	23 (18.3)	5 (2.6)	<0.0001
Tandem lesion ^‡^	39 (31.0)	26 (13.5)	0.0002
Lesion stenosis *, n (%)			
30–49	1 (0.8)	11 (5.7)	<0.0001
50–70	115 (91.3)	178 (92.2)	
71–90	10 (7.9)	4 (2.1)	
Invasive physiologic index *			
Median FFR value	0.75 (0.70, 0.78)	0.88 (0.84, 0.91)	<0.0001
Median NTG-Pd/Pa	0.80 (0.74, 0.84)	0.92 (0.88, 0.95)	<0.0001
Treatment strategy *, n (%)			
Revascularization	114 (90.5)	1 (0.5)	NA
Medical therapy alone	12 (9.5)	192 (99.5)	
Median time to first event (yr)	2.59 (1.44, 3.60)	3.22 (2.15, 4.46)	0.002

Abbreviations: CAD, coronary artery disease; CKD, chronic kidney disease; FFR, functional flow reserve; HbA1C, hemoglobin A1c; LDL, low density lipoprotein; LVEF, left ventricular ejection fraction; NTG-Pd/Pa, nitroglycerine-induced acute drop of mean distal pressure/mean proximal pressure; NTUH, National Taiwan University Hospital. * Count as per vessel, otherwise count as per patient. ^†^ A diffuse lesion was defined as a stenosis involving more than one segment. ^‡^ A tandem lesion was defined as two separate stenoses in the same coronary artery, separated by an angiographically normal segment.

**Table 2:** Medication at discharge and revascularization strategy of patients with CKD who received FFR examination at NTUH, stratified by FFR ≤ 0.8 (revascularization) and FFR > 0.8 (non-revascularization).

Demographic	Revascularization (*n* = 106 Patients)	Non-Revascularization (*n* = 136 Patients)	*p*-Value
Medication at discharge, n (%)			
Aspirin or P2Y12 inhibitor	97 (91.5)	117 (86.0)	0.186
Statin	56 (52.8)	75 (55.1)	0.720
Beta-blocker	67 (63.2)	81 (59.6)	0.563
Revascularization strategy *, n (%)			
Coronary artery bypass grafting	2 (1.6)	0(0)	-
Percutaneous coronary intervention	112 (88.9)	1 ^†^ (0.5)	
Drug-eluting stent or bioresorbable vascular scaffold	71 (63.4)	1 ^†^ (100)	
Bare metal stent	33 (29.5)	0 (0)	
Drug-coated balloon or plain old balloon angioplasty only	8 (7.1)	0 (0)	

Abbreviations: CKD, chronic kidney disease; FFR, functional flow reserve; NTUH, National Taiwan University Hospital. * Count as per vessel, otherwise count as per patient. ^†^ The operator decided to put a DES stent at the stenosis according to the inducible ischemia at myocardial perfusion imaging, though the FFR value showed 0.82.

**Table 3:** Incidence of clinical outcomes and adjusted hazard ratio of FFR-guided treatment strategy in a CKD population.

	Revascularization		Non-Revascularization		Crude HR (95% CI)	*p*-Value	Adjusted HR * (95% CI)	*p*-Value
	Total Number of Events (%)	Incidence Rate (Per 100 Person-Years)	Total Number of Events (%)	Incidence Rate (Per 100 Person-Years)				
Composite outcome	27 (25.5)	9.03	18 (13.2)	3.84	2.32 (1.28–4.24)	0.006	2.06 (1.07–3.97)	0.030
Death from any cause	31 (29.3)	10.4	33 (24.3)	7.04	1.54 (0.94–2.52)	0.086	1.67 (0.95–2.92)	0.073
Cardiac death	9 (8.5)	3.01	8 (5.9)	1.71	1.78 (0.69–4.64)	0.236	1.97 (0.69–5.60)	0.203
Non-fatal MI	3 (2.8)	1.00	2 (1.5)	0.43	2.32 (0.38–14.0)	0.359	2.61 (0.26–26.0)	0.413
Cardiac death + non-fatal MI	12 (11.3)	4.02	10 (7.4)	2.13	1.89 (0.81–4.39)	0.138	2.20 (0.89–5.43)	0.089
TVF *	22 (17.5)	6.34	16 (8.3)	2.46	2.53 (1.33–4.82)	0.005	2.19 (1.10–4.37)	0.026

Abbreviations: CI, confidence interval; CKD, chronic kidney disease; FFR, functional flow reserve; HR, hazard ratio; MI, myocardial infarction; TVF, target vessel failure. * Count as per vessel, otherwise count as per patient.

**Table 4:** Incidence of the composite outcome and hazard ratio of FFR-guided treatment strategy in CKD population, stratified by mild CKD, moderate to advanced CKD and dialysis dependent CKD.

	Revascularization		Non-Revascularization		HR (95% CI)	
	Total Number of Events (%)	Incidence Rate (Per 100 Person-Years)	Total Number of Events (%)	Incidence Rate (Per 100 Person-Years)		Interaction *p*-Value
Mild CKD	9 (20.9)	0.14	6 (9.7)	0.038	2.49 (0.88–7.03)	0.540
Moderate to advanced CKD	4 (17.4)	0.31	5 (10.4)	0.066	2.45 (0.65–9.24)	
Dialysis dependent CKD	14 (35)	0.40	7 (26.9)	0.47	1.13 (0.45–2.83)	

Abbreviations: CI, confidence interval; CKD, chronic kidney disease; FFR, functional flow reserve; HR, hazard ratio.

## Data Availability

Data cannot be shared publicly due to ethical restrictions. Data are available from the Integrated Medical Database of National Taiwan University Hospital (NTUH-iMD) for researchers who meet the criteria for access by review of our Institutional Review Board. For more information about data access, please contact the Department of Medical Research of National Taiwan University Hospital (030969@ntuh.gov.tw).

## Supplementary Materials

The following are available online at https://www.mdpi.com/article/10.3390/jpm12010021/s1, Table S1: Incidence of the clinical outcomes and crude hazard ratio of different revascularization strategies in functional ischemia group.
