# Supplementary material for: Long-Term Clinical Outcomes of Fractional Flow Reserve-Guided Coronary Artery Revascularization in Chronic Kidney Disease
Source: J Pers Med. 2022 Jan 1;12(1):21. doi: 10.3390/jpm12010021 (PMC8781197; doi:10.3390/jpm12010021)
Supplement: Supplementary file 1 [file jpm-12-00021-s001.zip › jpm-1448080-supplementary.pdf]

Supplemental material

Table S1. Incidence of the clinical outcomes and crude hazard ratio of different revascularization strategies in functional ischemia group.

	**DES or BVS**		BMS or POBA*		Crude HR (95% CI)	P-value
	**Total number of events (%)**	Incidence rate (per 100 person-years)	Total number of events (%)	Incidence rate (per 100 person-years)		
**Composite outcome**	**13/62 (21.0)**	0.11	10/31 (32.3)	0.49	0.47 (0.21 – 1.09)	0.078
TVF	9/71 (12.7)	0.057	11/41 (26.8)	0.30	0.35 (0.14 – 0.85)	0.020

Abbreviations: BMS: Bare metal stent; BVS: bioresorbable vascular scaffold; DES: Drug-eluting stent; POBA: plain old balloon angioplasty; TVF: target vessel failure. * Including revascularization with BMS, POBA and drug-eluting balloon.
